# Novel Biomarkers and Advanced Imaging in Cardiovascular Risk Stratification for Rheumatic Diseases

**DOI:** 10.7759/cureus.89794

**Published:** 2025-08-11

**Authors:** Freya H Shah, Siddharth Agrawal, Ritu C Tated, Darshilkumar Maheta, Syed Naqvi

**Affiliations:** 1 Internal Medicine, Landmark Medical Center, Woonsocket, USA; 2 Internal Medicine, Smt. Nathiba Hargovandas Lakhmichand (NHL) Municipal Medical College, Ahmedabad, IND; 3 Public Health, New York Medical College, Valhalla, USA

**Keywords:** ct scan and advance imaging, inflammation, novel biomarkers, rheumatic disorder, risk stratification

## Abstract

Rheumatic diseases, such as rheumatoid arthritis (RA) and systemic lupus erythematosus (SLE), significantly increase the risk of cardiovascular disease (CVD), with affected patients exhibiting a 2-3-fold higher risk compared to the general population. This elevated risk is primarily driven by chronic inflammation and accelerated atherosclerosis, which are not fully captured by traditional cardiovascular risk calculators. RA patients face cardiovascular risks similar to those of type 2 diabetics, while SLE patients, particularly young women, have a dramatically increased risk of myocardial infarction. These conditions challenge the conventional understanding of CVD risk, as traditional factors like hypertension, dyslipidemia, and smoking do not fully account for the excess risk observed. This has led to growing interest in the use of new biomarkers and advanced imaging technologies to improve risk stratification and early detection of cardiovascular involvement.

This review examines the pathophysiological mechanisms that link rheumatic diseases with cardiovascular risk. Chronic inflammation, immune dysregulation, and vascular dysfunction are central to the accelerated atherosclerosis and myocardial damage seen in RA and SLE. Key proinflammatory cytokines, including Tumor Necrosis Factor- Alpha (TNF-α), Interleukin (IL) IL-6, and IL-17, contribute to endothelial dysfunction and oxidative stress, exacerbating cardiovascular risk. In addition, biomarkers such as high-sensitivity C-reactive protein (hs-CRP), NT-proBNP, and troponins are valuable for detecting subclinical cardiac involvement and predicting adverse cardiovascular outcomes. Emerging biomarkers like IL-32, Dickkopf-related protein 1 (DKK-1), and galectin-3 also show potential in further refining risk assessment, particularly for atherosclerosis and myocardial fibrosis in rheumatic diseases.

Advanced imaging methods, such as transthoracic echocardiography (TTE), carotid ultrasonography, cardiac MRI (CMR), and coronary CT angiography (CCTA), provide key insights into subclinical cardiovascular changes in rheumatic disease patients. These techniques enable the detection of myocardial inflammation, fibrosis, and early atherosclerosis, helping guide clinical decisions and preventive interventions.

Despite advancements, traditional cardiovascular risk calculators often underestimate CVD risk in rheumatic disease patients, leading to the use of adjusted models, such as EULAR-endorsed 1.5× risk multiplier for RA patients. These adjustments, along with the integration of biomarkers and imaging findings, can help identify high-risk individuals and prompt early interventions, such as statin therapy. However, challenges remain, including the cost and accessibility of some imaging methods, the heterogeneous risk profiles across different rheumatic diseases, and the need for more prospective trials to evaluate the effectiveness of biomarkers and imaging in clinical practice.

In conclusion, incorporating biomarkers and advanced imaging techniques into cardiovascular risk assessment provides a more accurate method for managing CVD in rheumatic disease patients. These approaches allow for more personalized care, helping reduce the increased CVD mortality seen in this population. Future research should focus on refining multi-biomarker algorithms, improving imaging technology, and conducting intervention trials to optimize cardiovascular outcomes in rheumatic disease patients.

## Introduction and background

Rheumatic disease patients, particularly those with rheumatoid arthritis (RA), face a 2-3-fold increased risk of cardiovascular disease (CVD) compared to the general population, primarily due to chronic inflammation and accelerated atherosclerosis [1]. Traditional risk stratification tools, such as the Framingham Risk Score and ASCVD risk algorithm, underestimate CVD risk in these patients. New biomarkers and imaging modalities offer promising avenues for improved risk stratification and early treatment [2]. Systemic lupus erythematosus (SLE) presents an even greater risk, especially among young women. The excess risk attributable to rheumatic diseases is not explained by the traditional CVD risk factors of hypertension, dyslipidemia, and smoking [3]. In addition to RA and SLE, several other inflammatory rheumatic diseases - including psoriatic arthritis (PsA), ankylosing spondylitis (AS), and juvenile idiopathic arthritis (JIA) - have also been linked to increased cardiovascular risk. However, the strength and consistency of evidence vary among these conditions [4]. In order to identify subclinical cardiovascular involvement and enhance risk stratification, there has been a raised interest in new biomarkers and cutting-edge imaging methods. Because it allows for the prompt adoption of preventive measures (active risk factor control and optimal rheumatic disease therapy), early identification of high-risk individuals is essential. The pathophysiological connections between CVD and rheumatic disorders are thoroughly reviewed in this review, which also addresses the function of new biomarkers and state-of-the-art cardiovascular imaging techniques in adult and, when available, pediatric populations. This narrative review discusses the pathophysiological connections between CVD and rheumatic disorders, focusing on the role of chronic inflammation, immune dysregulation, and endothelial dysfunction. Key sections include emerging biomarkers, advanced imaging tools, limitations of current risk models, and clinical risk stratification approaches.

While many of these tools are promising, their real-world implementation remains limited due to cost, accessibility, and lack of standardized guidelines, especially in low-resource settings.

## Review

Pathophysiological link between rheumatic disease and cardiovascular risk

Chronic systemic inflammation drives endothelial dysfunction, oxidative stress, and lipid metabolism alterations, while autoimmune mechanisms exacerbate plaque formation and instability. Proinflammatory cytokines, including tumor necrosis factor-alpha (TNF-α), interleukin-6 (IL-6), and interleukin-17 (IL-17), are elevated in rheumatic disorders due to persistent inflammation [5]. These cytokines cause direct damage to the vascular endothelium and encourage atherosclerosis. In RA, endothelial dysfunction and cardiovascular death are correlated with C-reactive protein (CRP), a measure of systemic inflammation [6]. By increasing the expression of adhesion molecules (such as VCAM-1 and ICAM-1), these cytokines promote plaque formation and leukocyte infiltration into artery walls [6, 7]. Similarly, SLE patients have a much higher risk of CVD; vasculitis, immunological complex deposition, and inflammation can impact the myocardium and coronary arteries [8]. Young SLE women experience early coronary artery disease; a seminal study found that the risk of Myocardial Infarction (MI) in SLE women under 45 was more than 50 times greater. A second risk factor for arterial thromboses and ischemic events is SLE-specific, such as antiphospholipid antibodies (in antiphospholipid syndrome). Chronic inflammation and long-term corticosteroid usage contribute to metabolic syndrome traits, which is one reason why traditional risk factors (such as hyperlipidemia and hypertension) frequently cluster in rheumatic patients as well. Nevertheless, even after controlling for these factors, an inflamed vasculature retains a higher baseline risk.

In recent years, a growing body of research has explored the utility of emerging biomarkers to enhance cardiovascular risk stratification in patients with rheumatic diseases. Biomarkers such as NT-proBNP, Galectin-3, interleukin-32 (IL-32), Dickkopf-1 (DKK-1), and high-sensitivity cardiac troponins have shown promise in detecting subclinical myocardial dysfunction, vascular inflammation, and fibrotic remodeling features that often go unnoticed in traditional risk models. By integrating these markers with clinical parameters, clinicians may be better equipped to identify high-risk individuals and guide personalized therapeutic decisions.

Emerging biomarkers in cardiovascular risk assessment

The potential of several circulating biomarkers to improve cardiovascular risk assessment in rheumatic disorders has been studied. These include cytokines, new molecules implicated in the pathophysiology of atherosclerosis and rheumatic illness, general indicators of inflammation, and markers of cardiac-specific harm. Combining multiple biomarkers may improve cardiovascular risk prediction over single markers. Multi-marker panels incorporating hsCRP, adiponectin, osteoprotegerin, SAA, and IL-6 have shown greater accuracy in predicting subclinical vascular inflammation and cardiovascular events in rheumatic populations. Such algorithms may support more personalized risk stratification in the future. We go over important biomarkers and their significance below:

*C-reactive Protein (*CRP*)*

Acute-phase reactant high-sensitivity C-reactive protein (hsCRP) is increased in psoriatic arthritis, active RA, and other inflammatory diseases. In both the general population and RA, elevated baseline CRP has been repeatedly linked to increased future risk for CVD [9]. Beyond conventional risk variables, CRP levels in RA patients can increase risk; according to one study, every 20 mg/L rise in CRP was linked to a ~1% increase in the absolute risk of a 10-year cardiovascular event. An elevated hsCRP over time indicates persistent inflammation, which might directly encourage the development of plaque [10]. As a result, hsCRP is a useful tool for identifying rheumatoid arthritis patients who would require vigorous cardiovascular prophylaxis in addition to being a disease activity measure. However, CRP is a non-specific marker and may be elevated due to infections, flares, or metabolic conditions, which limits its specificity for cardiovascular risk stratification in rheumatic disease patients. In psoriatic arthritis (PsA), elevated CRP and erythrocyte sedimentation rate (ESR) have been linked to increased carotid intima-media thickness and endothelial dysfunction. In ankylosing spondylitis (AS), CRP correlates with disease activity, but its association with cardiovascular events is less well defined.

N-terminal pro-B-type Natriuretic Peptide (NT-proBNP)

A sensitive indicator of ventricular dysfunction, NT-proBNP is produced by the ventricular myocardium in response to wall stress [11]. NT-proBNP may be somewhat high in rheumatic individuals who do not have heart failure because of subclinical myocarditis or mild ventricular diastolic dysfunction. It has been demonstrated that NT-proBNP levels are greater in RA patients than in healthy controls, and crucially, a higher NT-proBNP level in RA is linked to a poorer prognosis [12]. About 39% of a group of patients with early inflammatory arthritis had NT-proBNP ≥100 pg/mL; these patients also had a higher incidence of carotid plaque and a considerably higher 10-year death rate from cardiovascular and all causes. In the RA cohort, an NT-proBNP level of ≥100 pg/mL was associated with a roughly 3.4-fold higher risk of CV mortality. According to these findings, NT-proBNP may be used as a biomarker to reveal hidden cardiac involvement (such as asymptomatic left ventricular dysfunction) and pinpoint individuals who may benefit from cardioprotective measures [13]. NT-proBNP may also guide early cardiac imaging in patients with persistent elevation, even in the absence of overt heart failure. However, its interpretation may be confounded by factors like age, renal impairment, and comorbidities.

Cardiac Troponins (High-Sensitivity Tests)

Minute amounts of myocardial damage can be detected with high-sensitivity troponin T or I (hs-cTnT/I) assays [14]. Hs-troponin levels can occasionally be found to be elevated in patients with active rheumatic illness, even in the absence of obvious cardiac symptoms. This might be a sign of persistent subclinical cardiac damage from microinfarctions or inflammatory myocarditis [15]. Studies on RA have shown that individuals with high disease activity had somewhat higher levels of hs-troponin, which correlates with TNF levels and RA disease activity (such as swollen joint count, DAS28). Six percent of asymptomatic SLE patients had hs-cTnT levels above the 99th percentile, which is associated with cardiac MRI (T2 mapping) myocardial edema. Elevated hs-cTnT in RA patients is associated with TNF-α levels and disease activity (DAS28-ESR, r = 0.47) [16]. The sensitivity for myocardial damage in lupus nephritis is 85.7% when the threshold for hs-cTnT is set at 3 ng/L [15]. Detection of elevated high-sensitivity troponins in asymptomatic patients may prompt cardiology referral for imaging and closer monitoring for myocarditis or microvascular ischemia. However, levels can also rise due to chronic kidney disease, systemic inflammation, or assay variability, requiring careful interpretation to avoid overdiagnosis.

IL-32, or Interleukin-32

IL-32 contributes to vascular inflammation by inducing IL-6 and TNF-α, enhancing endothelial dysfunction and pro-atherogenic lipid changes. Proinflammatory cytokine IL-32 is increased in RA and SLE, where it affects atherosclerotic plaque instability and endothelial dysfunction [17]. Mechanistically, IL-32 stimulates the production of interleukin-6 (IL-6) and tumor necrosis factor-α (TNF-α) that exacerbates vascular inflammation and decreases high-density lipoprotein (HDL) cholesterol levels. Regardless of conventional risk factors, IL-32 is associated with elevated carotid intima-media thickness (CIMT) and arterial stiffness in RA patients [18]. Changed lipid profiles, like elevated HDL cholesterol in RA groups, are associated with a functional promoter single-nucleotide polymorphism (rs4786370) in the IL32 gene, suggesting involvement in inflammation and lipid metabolism. These results demonstrate IL-32 to be a biomarker and CVD modulator in rheumatic diseases [19]. High IL-32 expression may support the decision to initiate or escalate anti-inflammatory treatment in RA patients with increased vascular risk. The IL-32 polymorphism may also hold prognostic value, though further studies are needed to confirm its clinical utility. Additionally, the lack of assay standardization and the influence of overall inflammation present interpretive challenges.

Dickkopf-1 (DKK-1)

In RA, DKK-1, a Wnt signaling pathway inhibitor, enhances vascular calcification and synovial inflammation [20]. Intravascular ultrasonography studies have linked DKK-1 to unstable coronary plaques, and elevated circulating levels of DKK-1 predict cardiovascular mortality in RA patients [hazard ratio (HR): 2.1, 95% confidence interval (CI): 1.4-3.2] [14]. Ironically, DKK-1 is associated positively with aortic valve calcification but inversely with diffuse vascular calcification, indicating that its function in mineral metabolism varies with context [21]. DKK-1 can be a therapeutic target in unstable atherosclerotic plaques since it promotes inflammatory cell infiltration and suppresses beneficial calcification [21]. Elevated DKK-1 levels may help identify patients with unstable plaque features and could support early initiation of statins or anti-inflammatory therapy. However, its dual roles in promoting and inhibiting different types of calcification, combined with the lack of validated clinical thresholds, make routine use challenging at present.

Galectin-3 (Gal-3)

Galectin-3 is a β-galactoside-binding lectin that promotes myocardial fibrosis by stimulating macrophages and cardiac fibroblasts. Myocardial dysfunction and vascular fibrosis are associated with increased levels of galectin-3, a mediator of fibrosis and immunological dysregulation, in RA and SLE. In rheumatic cohorts, serum Gal-3 levels more than 12.5 ng/mL are predictive of myocardial fibrosis on cardiac magnetic resonance imaging (CMR) and hospitalization for heart failure [22]. Regardless of conventional cardiovascular risk factors, Gal-3 is linked to arterial stiffness, systemic vascular resistance, and decreased cardiac output in RA. These results imply that Gal-3 is a viable biomarker for subclinical CVD as it combines inflammatory and fibrotic pathways [23]. Galectin-3 may guide decisions regarding cardiac imaging or intensification of immunosuppression in patients with suspected myocardial fibrosis. However, its levels may be affected by renal function and lack disease-specific cut-offs, limiting its current clinical use.

While hsCRP and troponins remain the most widely used markers in clinical practice, emerging biomarkers such as IL-32 and Galectin-3 may detect early vascular changes and fibrosis not captured by traditional markers. However, limited availability, lack of thresholds, and confounding factors currently restrict their use beyond research settings. Other biomarkers such as catestatin, fetuin-A, asymmetric dimethylarginine (ADMA), and circulating miRNAs are currently being explored in broader cardiovascular contexts. Despite their promise, circulating microRNAs face challenges related to assay variability, sample processing differences, and lack of standardized cut-offs, which currently limit their clinical settings.

Cytokines and Immune Mediators

Circulating cytokines, including TNF-α, IL-1β, and IL-6, have been studied as indicators of CV risk and are increased in active RA and SLE. Particularly, IL-6 is highly pro-atherogenic; elevated IL-6 levels are linked to arterial inflammation and endothelial dysfunction [3]. IL-6 is independently associated with carotid plaque in RA patients. Similarly, endothelial activation indicators such as E-selectin and soluble vascular cell adhesion molecule-1 (sVCAM-1) are frequently increased in RA and are associated with carotid intima-media thickness (cIMT) and coronary artery calcium scores [10]. These cytokines support the link between inflammation and atherosclerosis, although they are not frequently tested in practice.

In RA, there is a positive correlation between Cytokines and Immune Mediators IL-6 and the Framingham Risk Score (HR: 1.57, 95% CI: 1.44-1.72) [24] and SCORE (HR: 1.42, 95% CI: 1.31-1.53). Arterial inflammation is decreased by IL-6 inhibitors (ΔTBR = -0.21) [25].

Anti-Cyclic Citrullinated Peptide (CCP) Antibodies

In RA, increased carotid intima-media thickness (CIMT) is associated with left ventricular dysfunction, with a significant decrease in LVEF (P = 0.01) [26]. Anti-CCP antibodies may contribute to vascular dysfunction through direct endothelial cell activation and induction of citrullinated protein-targeted inflammation, promoting atherosclerotic plaque instability and microvascular injury.

The endogenous peptide catestatin, which regulates cardiovascular function, and the calcification inhibitor fetuin-A, which has been linked to cardiovascular risk profiles in RA [23], are other options. Although these new biomarkers are not yet clinically available, they are a prime example of the multi-biomarker panel trend. Furthermore, research is being done on microRNAs (small non-coding RNAs) that control gene expression; certain circulating microRNA profiles (such as miR-146a and miR-155) have been connected to RA's inflammation and atherosclerosis and may be used as biomarkers in the future [23].

Lastly, indicators of endothelial function (asymmetric dimethylarginine, ADMA), oxidative stress (like myeloperoxidase), and lipid composition (such as defective HDL or lipoprotein(a)) are also being investigated for their role in cardiovascular risk in rheumatology [10]. The most reliable risk categorization for these individuals may be provided by algorithms that combine conventional parameters with specialized biomarkers (inflammatory and cardiac-specific) as our knowledge advances.

Panels with Multiple Biomarkers

Because no single biomarker fully captures the multifactorial nature of cardiovascular risk in rheumatic diseases, integrated panels offer a more robust approach by incorporating inflammatory, metabolic, and fibrotic markers. Beyond conventional methods, integrated biomarker panels improve the prediction of CVD risk in RA. Six biomarkers - serum amyloid A (SAA), C-reactive protein (CRP), soluble TNF receptor 1 (sTNFR1), adiponectin, YKL-40, and osteoprotegerin - were shown in a 2024 cohort study to enhance the prediction of vascular inflammation (ΔR2 = +0.13) when combined with clinical factors. These findings require external validation in larger, ethnically diverse populations to ensure generalizability and clinical translation [27]. These biomarkers allow for customized risk classification by reflecting a variety of processes, such as vascular remodeling (YKL-40), metabolic dysregulation (adiponectin), and inflammation (SAA, CRP) [28]. CRP, SAA, and YKL-40 often reflect overlapping inflammatory pathways, and their redundancy may limit individual predictive power; however, composite biomarker panels can harness complementary kinetics and improve risk discrimination. Implementation of multi-biomarker algorithms in routine care faces logistical barriers, including cost, need for specialized assays, lack of harmonized reporting standards, and integration with existing rheumatology-cardiology workflows.

Advanced imaging methods

In individuals with rheumatic illnesses, advanced cardiovascular imaging has emerged as a crucial supplementary tool for identifying subclinical heart and vascular disease. This is especially relevant in RD populations, where chronic systemic inflammation, autoimmune-mediated vascular injury, and atypical symptom presentation often lead to silent or early-onset cardiovascular involvement that may not be detected by traditional risk screening or routine clinical assessment. Imaging can indicate quiet organ involvement that traditional risk assessment might overlook. Nowadays, a variety of noninvasive imaging techniques are available, each with special advantages for describing the anatomy and function of the heart. Here, we go over the primary imaging methods and how they are used to treat rheumatic diseases. Cardiac MRI (CMR) is preferred for assessing myocardial inflammation and fibrosis; PET-CT is optimal for detecting active vascular inflammation; and CCTA is best for evaluating coronary plaque burden, especially in patients with atypical symptoms or low-intermediate pre-test probability.

Transthoracic echocardiography (TTE) is a commonly accessible, first-line method for evaluating the structure and function of the heart. Echocardiography can identify abnormalities in inflammatory rheumatic illnesses that patients may not yet be exhibiting symptoms of, such as diastolic dysfunction, pulmonary hypertension, or valvular lesions [29]. For instance, a lower E/A ratio on Doppler echo or greater filling pressures are indicators of asymptomatic left ventricular diastolic dysfunction, which is more common in RA and SLE patients than in healthy people. Up to 30% of SLE patients have valvular thickening and nodules (Libman-Sacks endocarditis) on echo, even if they are clinically quiet [30]. The sensitivity of advanced echocardiographic methods has been considerably enhanced. One sensitive metric of myocardial systolic function is global longitudinal strain, which may be measured via speckle-tracking echocardiography (STE). Global longitudinal strain (GLS) detects subtle myocardial dysfunction even when ejection fraction remains normal, making it valuable for identifying preclinical cardiomyopathy in rheumatic patients.

Additionally, ultrasound is used to assess vascular health, most frequently using carotid ultrasonography. Carotid plaque detection and carotid intima-media thickness (cIMT) measurement are well-established methods for assessing subclinical atherosclerosis. In rheumatic illnesses, when atherosclerosis is increased, they are extremely important. Research continuously demonstrates that compared to age- and risk-factor-matched controls, RA patients have higher cIMT and a higher incidence of carotid plaque. In identifying which RA patients are likely to have cardiac incidents, carotid ultrasonography may perform better than conventional risk evaluations. This has led to some professional advice to use carotid ultrasonography in inflammatory arthritis risk assessment. For example, the European Alliance of Associations for Rheumatology (EULAR) advises that if an ultrasound reveals any carotid plaque, a patient with RA who has a borderline risk score should be upgraded to a high-risk group, requiring preventative medication [31]. Carotid ultrasonography is a practical, radiation-free method that may be used repeatedly to track the development of atherosclerosis. Even though the patient's conventional risk factors alone would not suggest it, a doctor may decide to start statin therapy or increase anti-inflammatory treatment if they observe plaque or high IMT in a patient with lupus or RA.

Cardiac MRI (CMR) is the gold standard for diagnosing cardiomyopathy and myocarditis and provides unmatched images for characterizing cardiac tissue [32]. The capacity to identify myocardial inflammation, fibrosis, and perfusion abnormalities, even in the absence of symptoms, makes it relevant in rheumatic illnesses. Systematic reviews and meta-analyses comparing SLE patients with matched controls reveal significant differences in cardiac structure and function. SLE patients have higher indexed end-diastolic volume (77 vs. 72 mL/m^2^, p=0.006) and a slightly but significantly worse left ventricular ejection fraction (62% vs. 64%, p=0.001). Additionally, late gadolinium enhancement (LGE) burden is larger in SLE patients, suggesting myocardial scarring (3.5% vs. 1.1% of LV mass, p=0.009) [33].

Diffuse myocardial inflammation and fibrosis are indicated by the considerably increased native T1 and T2 relaxation periods in SLE patients when compared to controls (native T1 [1.5T]: 1005 vs. 982 ms, p=0.02; native T1 [3T]: 1267 vs. 1140 ms, p<0.001; T2: 58 vs. 51 ms, p<0.001). Three studies have found a correlation between elevated T2 relaxation periods and SLE disease activity, indicating that these parameters are correlated with disease activity. SLE can subclinically affect the heart muscle (lupus myocarditis) [34]. T2-weighted CMR sequences can detect myocardial edema, or inflammation, and localized fibrosis or scarring can be seen with late gadolinium enhancement (LGE) in both localized and diffuse [35]. Interestingly, compared to individuals with long-standing SLE, those with new-onset SLE have more marked myocardial edema (higher T2 values), indicating more active inflammation in the early stages of the disease [36]. In autoimmune illnesses, these alterations frequently occur before overt cardiac failure, which makes this crucial [35]. Comparing RA patients to controls, the former show increased T1, extracellular volume (ECV), and T2s [37]. According to one study, LGE was detected in 46% of RA patients compared to none in controls [38]. The majority of these patients had patchy mid-wall enhancement, a non-ischemic pattern. These results suggest that traditional measurements of cardiac function could overlook early myocardial illness, even in the presence of a normal left ventricular ejection fraction.

Functional evaluation

CMR can measure exact biventricular volumes, mass, and ejection fraction. It can also evaluate mild myocardial strain deficits that echocardiography could miss.

Identification of subclinical disease

Research has demonstrated that CMR can identify myocardial anomalies in RA and SLE patients who do not exhibit cardiac symptoms. Perfusion imaging and coronary artery disease detection are other capabilities of CMR. Significant coronary microvascular impairment has been found in rheumatic individuals by CMR and SPECT investigations. In 44% of SLE patients with chest discomfort who did not have obstructive coronary artery disease, stress CMR revealed alterations in perfusion. According to SPECT investigations, 50% of SLE patients had perfusion deficiencies, and 35% of such patients experienced associated symptoms [39] who had never had coronary artery disease before displayed [40]. These results highlight the possibility of serious ischemic heart disease in young rheumatic patients, which can only be identified by sophisticated imaging.

Coronary CT angiography (CCTA) and calcium scoring have become critical tools for assessing cardiovascular risk in rheumatoid arthritis (RA) patients, revealing insights into atherosclerotic burden and plaque vulnerability driven by chronic inflammation.

According to numerous studies, 10.3% of controls had severe calcification (Coronary artery calcium [CAC] > 400), whereas 14.9% of RA patients had it, and 59.7% of RA patients and 50.0% of non-RA controls, respectively, had a CAC score higher than zero [41]. A greater load of calcified coronary plaque than in the general population is indicated by the almost 60% of RA patients who had elevated CAC scores, according to other studies and meta-analyses [42]. Additionally, RA patients have higher mean CAC scores than controls (e.g., mean CAC of 246.8 in RA vs. 0.6 in controls in one research) and more high-risk plaque characteristics that include non-calcified plaques and multi-vessel [43]. Regardless of conventional cardiovascular risk factors, this elevated plaque load and susceptibility are observed even in RA patients who do not exhibit symptoms and are linked to increased disease activity [41].

A rightward shift in the arterial age distribution and a noticeably greater likelihood of having a nonzero CAC score than matched controls are indicators of accelerated arterial aging in RAs [44]. A high CAC score unmistakably indicates the need for early preventive measures, such as statin medication, even while a zero CAC score may not completely rule out risk in RA, since non-calcified, high-risk plaques may still be present. In order to provide more proactive cardiovascular prevention and therapy, our data suggest the use of CAC score and CCTA in a subset of RA patients, particularly those with symptoms or high-risk profiles [41, 43]. Unlike coronary artery calcium (CAC) scoring, which only quantifies calcified plaque, CCTA also detects non-calcified, high-risk plaques and provides anatomical localization, making it superior for comprehensive coronary risk assessment.

One of the most important tools for observing inflammatory and fibrotic processes in the cardiovascular consequences of rheumatic disorders is Positron Emission Tomography Combined with Computed Tomography (PET-CT). About 53.1% of RA patients have carotid plaque, which is significantly higher than the 37.0% seen in age/sex-matched controls (p = 0.038), according to studies using the glucose analog tracer ¹⁸F-fluorodeoxyglucose (FDG). The prevalence of plaque is also independently correlated with interleukin-6 (IL-6) levels (HR: 2.03 per quartile; 95% CI: 1.26-3.26) [3, 45]. FDG uptake in arterial walls, a sign of vascular inflammation, is higher in RA patients with rheumatoid nodules or anti-cyclic citrullinated peptide (anti-CCP) seropositivity and has a strong correlation with systemic inflammation markers such as high-sensitivity C-reactive protein (hs-CRP; r = 0.58, p = 0.04) [3]. Serial CMR or PET-CT imaging can monitor therapeutic response by tracking changes in myocardial inflammation or vascular uptake over time, particularly in patients on biologic agents or corticosteroids. While PET-CT has shown utility in detecting vascular inflammation, it is currently considered a research tool and is not routinely recommended by major cardiovascular or rheumatologic guidelines.

RA patients with moderate-to-high disease activity had baseline aortic FDG uptake standardized uptake values (SUV) of 1.83 ± 0.34; however, those who experienced remission following biologic therapy had higher post-treatment SUVmax (2.05 ± 0.32 vs. 1.79 ± 0.33 in non-remission patients, p = 0.002) [46]. In eight individuals with diffuse myocardial FDG uptake (median troponin 0.08 ng/mL) and decreased ejection fraction due to systemic lupus erythematosus (SLE), FDG-PET/CT has been used to diagnose myocarditis when conventional imaging was equivocal. In myocardial fibrosis (MF) associated with systemic sclerosis (SSc), fibroblast activity is shown by emerging tracers such as Ga-fibroblast activation protein inhibitor (FAPI), which targets fibrosis. FAPI-PET/CT revealed greater tracer uptake associated with arrhythmias (p < 0.05), enhanced N-terminal pro-B-type natriuretic peptide (NT-proBNP; p < 0.01), and late gadolinium enhancement in six SSc patients with MF. Compared to FDG-PET/CT, FAPI-PET/CT identified 13.2% more organs implicated in IgG4-related illness, especially in the bile duct and pancreas. Studies showing a 40.1% improvement in net reclassification of cardiovascular risk beyond Framingham risk scores provide evidence of the predictive utility of FDG-PET/CT [47], despite its spatial resolution limitations for coronary imaging. By facilitating direct monitoring of anti-inflammatory treatments and early diagnosis of high-risk phenotypes like non-calcified plaques in RA (73.3% vs. 20% in controls, p = 0.04) or subclinical myocarditis in SLE [48, 49], these developments underscore PET-CT's dual function in research and clinical practice. Radiation exposure, expense, and the requirement for uniform quantification techniques among tracers such as FDG and FAPI are still issues, though [46].

CMR and PET-CT face limitations in routine practice due to high cost, limited availability, need for trained personnel, and long acquisition times, particularly in low-resource settings.

Clinical risk stratification

Currently, there are no universally accepted clinical guidelines that recommend integrating novel biomarkers or advanced imaging into CVD risk assessment algorithms for rheumatic disease patients, limiting their routine adoption in practice. Traditional cardiovascular risk calculators like Framingham and SCORE underestimate risk in rheumatic diseases due to unaccounted systemic inflammation. Adjustments like the EULAR-endorsed 1.5× multiplier for rheumatoid arthritis (RA) patients with prolonged disease duration or seropositivity are currently advised by recommendations to address this [50]. Using the new SCORE 2 model, for instance, this multiplier moved 30% of RA patients into higher risk groups. Risk stratification is further refined by imaging techniques such as carotid ultrasonography and coronary artery calcium (CAC) score [50]. Even if conventional scores indicate intermediate risk, early statin medication may be prompted by a CAC score > 400 in RA patients (14.9% vs. 10.3% in controls) or carotid plaque identification (73.3% in RA vs. 20% in controls). In RA, the addition of six biomarkers (e.g., serum amyloid A, osteoprotegerin) enhanced arterial inflammation prediction by 13% when compared to clinical factors alone [27]. Biomarkers, including hs-CRP, IL-6, and YKL-40, significantly improve prediction.

Limitations

Traditional models underestimate inflammation-driven risk, Framingham and SCORE do not account for inflammation-driven risk, as observed in RA patients with paradoxically low LDL but high atherosclerosis burden [51]. Accessibility and Cost: Advanced imaging (e.g., PET-CT, CMR) is still costly and restricted to research settings; for instance, FAPI-PET for fibrosis detection is not widely available despite its potential to identify early myocardial damage [51]; Biomarker Specificity: Elevated hs-CRP or troponin may reflect active arthritis or renal disease rather than cardiovascular risk, making interpretation more difficult; Heterogeneity: Risk profiles differ among rheumatic diseases (e.g., lupus vs. vasculitis) [52]; SLE-specific tools, such as SLECRISK, are still being developed [2]; Lack of Intervention Trials: The majority of the evidence is observational; there are few prospective studies testing statins or immunosuppressants guided by imaging/biomarkers [2]. Although IL-32, DKK-1, and galectin-3 show promise, they remain investigational and are not yet validated for routine clinical use due to limited prospective validation in diverse rheumatic populations.

## Conclusions

In rheumatic disorders, individualized cardiovascular risk management is made possible by combining inflammation-sensitive methods like CAC score, biomarkers (such as hs-CRP and IL-6), and sophisticated imaging. Adjustments such as the EULAR multiplier and imaging-guided categorization (e.g., carotid plaque detection) assist in identifying high-risk individuals for early intervention, even while standard models understate risk. High-risk categorization may guide early initiation of statins, tighter blood pressure control, escalation of immunosuppressive therapy, or referral for cardiology evaluation even in the absence of overt symptoms.

Cost, illness heterogeneity, and biomarker ambiguity are still obstacles, but new approaches like multi-biomarker algorithms and AI-enhanced imaging analysis hold promise. AI-enhanced imaging, such as machine learning algorithms applied to CCTA or CMR for plaque characterization or fibrosis quantification, is currently in research or early clinical implementation phases, with pilot models aiding automated detection of subclinical cardiac changes in rheumatic populations. Multi-biomarker algorithms may combine inflammatory markers (e.g., CRP, IL-6), cardiac stress markers (e.g., NT-proBNP, troponins), and vascular markers (e.g., Galectin-3, DKK-1) into a composite score, with recent studies suggesting improved predictive value over single markers. Findings such as elevated NT-proBNP or high-risk plaques on CCTA may prompt intensification of cardiovascular prevention strategies, including lipid-lowering, anti-inflammatory agents, or cardiac imaging surveillance. Proposed models for integrating rheumatology and cardiology include multidisciplinary clinics, shared care pathways, and EHR-linked risk dashboards that facilitate collaborative risk assessment and management. Ultimately, bridging rheumatology and cardiology through integrated risk models, biomarker-guided strategies, and tailored imaging holds the key to reducing the cardiovascular burden in rheumatic disease - an urgent frontier that now demands prospective validation and clinical action.
